# Project ECHO Dementia for PACE Programs: Strength in Partnerships and Lessons for the Future

**DOI:** 10.1093/geroni/igaf122.748

**Published:** 2025-12-31

**Authors:** Leland Waters, Phillip Clark, Faith Helm, Dana Sohmer, Jane Marks

**Affiliations:** Virginia Commonwealth University, Richmond, Virginia, United States; University of Rhode Island, Kingston, Rhode Island, United States; Rhode Island Geriatric Education Center, Kingston, Rhode Island, United States; Alzheimer’s Association, Chicago, Illinois, United States; Johns Hopkins University, Baltimore, Maryland, United States

## Abstract

Project ECHO Dementia has been a collaborative initiative designed to enhance dementia care. Over the past five years, three Geriatrics Workforce Enhancement Programs (GWEPs) have partnered with the Alzheimer’s Association National Office to implement two distinct Project ECHO series. This new series builds upon an established history of collaboration between GWEPs and regional Program of All-Inclusive Care for the Elderly (PACE) programs, which have actively participated in previous geriatrics education programs, including ECHO-based learning. Our ECHO framework follows the structured model with a dedicated interprofessional Hub team, fostering a collaborative “all teach, all learn” approach. PACE programs, already operating within an interprofessional care model, naturally align with this educational approach, leveraging team-based training to enhance dementia care. Through iterative development, we have adapted our program in response to participant feedback. Initially structured as twelve biweekly sessions, we transitioned to a more focused format of four weekly sessions based on a pre-series needs assessment. Key lessons learned include the importance of continuity of engagement among PACE learning sites and among subject matter experts to establish trust and rapport and the ability to build on previous sessions for progressive learning. We implemented a needs assessment to identify specific educational priorities for PACE programs, refining session content to enhance engagement and applicability and expanding interprofessional collaboration to strengthen dementia care training. Recommendations include maintaining continuity of subject matter experts, integrating participant feedback into future ECHO series, and exploring opportunities to scale and sustain the program for broader impact within geriatrics education.

